# Nanoporous Carbon Electrodes Derived from Coffee Side Streams for Supercapacitors in Aqueous Electrolytes

**DOI:** 10.3390/nano12152647

**Published:** 2022-08-01

**Authors:** Julian Selinger, Sebastian Stock, Werner Schlemmer, Mathias Hobisch, Nikolaos Kostoglou, Qamar Abbas, Oskar Paris, Christian Mitterer, Michael Hummel, Stefan Spirk

**Affiliations:** 1 Institute of Bioproducts and Paper Technology, Graz University of Technology, Inffeldgasse 23, 8010 Graz, Austria; julian.selinger@tugraz.at (J.S.); werner.schlemmer@ecolyte.at (W.S.); mathiashobisch@yahoo.com (M.H.); 2 Department of Bioproducts and Biosystems, Aalto University, P.O. Box 16300, 00076 Aalto, Finland; michael.hummel@aalto.fi; 3 Institute of Physics, Montanuniversität Leoben, Franz-Josef-Straße 18, 8700 Leoben, Austria; sebastian.stock@unileoben.ac.at (S.S.); oskar.paris@unileoben.ac.at (O.P.); 4 Department of Materials Science, Montanuniversität Leoben, Franz-Josef-Straße 18, 8700 Leoben, Austria; nikolaos.kostoglou@unileoben.ac.at (N.K.); christian.mitterer@unileoben.ac.at (C.M.); 5 Institute for Chemistry and Technology of Materials, Graz University of Technology, Stremayrgasse 9, 8010 Graz, Austria; qamar.abbas@tugraz.at; 6 Institute of Chemistry and Technical Chemistry, Faculty of Chemical Technology, Poznan University of Technology, Berdychowo 4, 60965 Poznan, Poland

**Keywords:** supercapacitor, ECDL, coffee waste, coffee silver skins, quinones, activated carbon, electrodes

## Abstract

Coffee, as one of the most traded resources, generates a vast amount of biogenic by-products. Coffee silver skins (CSS), a side stream from the roasting process, account for about 4 wt.%. Despite the abundancy of CSS, possible routes to generate added value for broad applications are limited. Herein, we present an approach to use CSS as a precursor material for supercapacitor electrodes. KOH activated carbon (AC) was produced from CSS. The resulting AC—CSS was characterized by X-ray diffraction, gas sorption analysis, scanning electron microscopy, and Raman spectroscopy. The highly porous AC—CSS exposes a specific surface area of more than 2500 m^2^ g^−1^. Electrodes formed with AC—CSS were electrochemically characterized by performing cyclic voltammetry and galvanostatic cycling. The electrodes were further assembled into a supercapacitor device and operated using 1 M sulfuric acid as electrolyte. In addition, various quinones were added to the electrolyte and their impact on the capacitance of AC—CSS electrodes was analyzed. In this work, we were able to show that CSS are a valuable source for supercapacitor applications and that coffee-waste-derived quinones can act as capacitance enhancers. Thus, the findings of this research show a valuable path towards sustainable and green energy storage solutions.

## 1. Introduction

Coffee beans are important commodities for the preparation of different types of coffee beverages. However, the production of coffee products involves many process steps, with each producing a wide range of waste materials. For instance, the harvesting of a single ton of green coffee beans results in ca. 0.5 tons of coffee pulp and 0.2 tons of husks [1,2]. In the course of the roasting process, CSS are obtained that cover the bean and peel off at elevated temperatures. Spent coffee grounds (SCG) are the most well-known side stream as they come from coffee brewing. CSS and SCG account for approximately 2% and 50%, respectively, in the weight of a dry coffee bean [2]. Although there are efforts to valorize these streams in, e.g., fertilizer applications, most of these materials are currently deposited at landfills or incinerated [3,4]. The composition of coffee silver skin, however, makes it a potentially promising material for various purposes. CSS features a significant lignin content (ca. 28% of the dry mass [5]), that could be exploited in applications where aromatic structures are beneficial. These comprise char formation and other processes where graphitic structures are required. Furthermore, due to its high protein content (ca. 18% [6]), it can also be a potential nitrogen source for applications where, e.g., the incorporation of heteroatoms in the carbon structure is desired. CSS also contains antioxidative components such as chlorogenic acid (CGA) and caffeic acid (CA). CGA and CA have also recently been associated with anti-inflammatory and anti-cancerogenic activity [7]. Their concentration, however, in the silver skins depends on the exact processing conditions. Thermal stress in the roasting process leads to decomposition reactions, reducing the amount of CGA and CA in the CSS. As CGA and CA are not readily water soluble, their extraction from the CSS requires the use of organic solvents such as ethanol, methanol, or mixtures thereof [8,9].

In recent years, the use of renewable materials for energy storage devices has seen a tremendous increase [10,11]. Therefore, precursor material is usually subjected to a physical (e.g., CO_2_, H_2_O) or chemical (e.g., ZnCl_2_, K_2_CO_3_, KOH) activation procedure. Both methods reveal advantages as well as disadvantages, in terms of yield, pore size distribution (PSD), surface area, costs, activation temperature, etc. Potassium hydroxide, as used in this study is known to be a powerful activation agent leading to highly specific surface areas. However, its commercial relevance is debatable [12]. The use of nanoporous carbon-based electrodes for supercapacitors has been described in several hundreds of papers during the last decade. While some of these materials offer interesting opportunities in the context of a biorefinery (e.g., bark materials, viscose fibers, nanocellulose [13,14,15,16]), most of the reported materials have only limited significance as only small volumes of raw material are available, impeding scale-up. For coffee-based carbons for supercapacitor applications, the focus has mainly been on spent coffee grounds, a readily available material that has shown satisfying performance characteristics in various setups. A potential drawback of SCG compared to CSS is its lower lignin content (SCG: ~16–24 wt.% vs. CSS ~28 wt.% [17]), which limits the theoretical carbon yield [18].

A recent trend to increase supercapacitor performance is to add redox active species into the electrolyte solution (for aqueous systems typically H_2_SO_4_ or KOH) [19,20]. Such species provide additional chemical storage via fast redox reactions in addition to the physical storage capacitance of a pure electrical double layer [21]. An important class of redox active components consists of hydroquinones that can be selectively oxidized to the corresponding quinones. While some quinones have been reported to enhance supercapacitor performance, CGA and CA as electrolyte additives in supercapacitor electrolytes have, to the best of our knowledge, not been reported so far. Therefore, in terms of charge storage in supercapacitors, CSS can be a valuable resource in two aspects: First, to improve supercapacitor electrolytes, where the redox active compounds (CA and CGA) can be extracted from CSS using well established procedures [8,9]. Second, the extraction residue can be subjected to carbonization and activation to obtain nanoporous biobased carbon for supercapacitor electrodes. 

Herein, we aim to combine different concepts for the creation of an “all-coffee-based supercapacitor device” by employing CSS as a carbon source for electrodes. Further, quinones which can potentially be extracted from coffee side streams, such as CA and pBQ (para-benzoquinone), among others, are employed as a redox mediator. This includes the preparation of freestanding supercapacitor electrodes, the assembly of a symmetric supercapacitor, and the characterization of the electrochemical performance at device level with and without CA addition.

## 2. Materials and Methods

Caffeic acid, predominantly trans ≥98%, was obtained from Alfa Aesar (Haverhill, MA, USA). Isopropanol (≥98%), and sulfuric acid (95%) were purchased from VWR Chemicals (Radnor, PA, USA). Super P from Timcal (Bodio, Switzerland), polytetrafluoroethylene (PTFE) (60 wt.% suspension in H_2_O; diluted to 10 wt.%) from Sigma-Aldrich (St. Louis, MO, USA) and YP—80F from Kuraray (Tokyo, Japan) were used. Glass microfiber filters (GF/A from Whatman, Maidstone UK), punched in 10 mm diameter, functioned as separator in a Swagelok^®^ T-cell, while LOCTITE^®^ EDAG PF 407C (Henkel, Düsseldorf, Germany) was used as conductive adhesive between electrodes and bolts. Para-benzoquinone (pBQ) (≥98%) was purchased from Alfa Aesar (Ward Hill, MA, USA). The synthesis of 2-methoxyhydroquinone (MHQ) was performed following the procedure described elsewhere [22]. Hydrochloric acid (37%) and potassium hydroxide (pellets; ground to powder using pestle and mortar) were purchased from Merck (Darmstadt, Germany). Coffee silver skins (ash content: 7.4%, dry content: 90.4%) from *Coffee Arabica* were obtained from a local company. 

### 2.1. Dry and Ash Content Determination 

The CSS was placed into Al_2_O_3_ crucibles and heated to 105 °C for 2 days to determine the dry content. Subsequently, it was further heated to 575 °C to obtain the ash content. 

### 2.2. Simultaneous Thermal Analysis (STA)

The STA measurements were performed on a Jupiter STA 449 F3 coupled with a mass spectrometer (MS) Aëolos QMS 403 (both Netzsch, Selb, Germany). Therefore, the sample (10 ± 1 mg) was heated under a constant synthetic air (20% oxygen, 80% nitrogen) or helium flow (100 mL min^−^^1^) from 40 °C towards the respective target temperature of 600 or 800 °C, respectively, with a heating rate of 10 K min^−1^.

### 2.3. Scanning Electron Microscopy (SEM)

SEM investigation was performed on a Sigma VP (Zeiss, Germany) at low acceleration voltage (0.7 kV) and detected with an in-lens detector. The CSS was coated by sputter deposition with a 4.0 nm layer of a platinum/palladium alloy (80/20), while the AC—CSS were analyzed without coating.

The energy-dispersive X-ray spectroscopy (EDX) signal was detected with an Ultim^®^ Max 65 (Oxford Instruments, Abingdon, UK) at 5.5 kV. The signal was further processed and evaluated in AZtecLive software.

### 2.4. Pre-Carbonization and Activation

CSS was dried at 105 °C for 24 h and allowed to cool to room temperature. Afterwards, the CSS (5 g) was put into an Al_2_O_3_ crucible (7 × 4.5 × 1.5 cm^3^), which was then placed into a three-zone tube furnace (TZF 15/610, Carbolite, Neuhausen, Germany). The heat treatment to obtain pre-carbonized material (PCM) was conducted under a constant nitrogen-flow (~0.5 L min^−^^1^). The target temperature (400 °C) was reached with a heating rate of 6 K min^−^^1^. After cooling to room temperature, KOH (5-fold excess, m/m) was added to the PCM and the mixture was ground by means of pestle and mortar for several minutes. Afterwards, the mixture was pyrolyzed (heating rate: 6 K min^−1^) at 800 °C for 120 min. After cooling to room temperature, the obtained material was dispersed in HCl (2 M, 100 mL) and stirred for 1 h using a magnetic stirrer (500 rpm). The powder was subsequently washed with an excess of deionized water until the used washing solution exhibited a neutral pH. The obtained activated CSS-based carbon (AC—CSS) was dried (24 h, 105 °C) and stored until further processing under exclusion of ambient atmosphere.

### 2.5. Gas Adsorption/Desorption Analysis

The low-pressure gas sorption experiment was conducted using a QuantaTech Autosorb iQ^3^ gas sorption analyzer (Boynton Beach, FL, USA) with argon (Ar) gas of ultra-high purity (99.999%). The AC—CSS (~50 mg) was degassed under vacuum (10^−6^ mbar) for 24 h at 250 °C prior to the measurement. The sample was placed in a glass cylinder cell and a glass filling rod was additionally placed inside the sample cell. The dead volume of the sample cell was automatically evaluated before each run using helium (He) gas of ultra-high purity (99.999%). Ar adsorption/desorption isotherms were recorded at 87.3 K in a relative pressure range (P/P_0_) from 10^−6^ to 0.99 in 77 steps for adsorption and 36 steps for desorption. To ensure a constant temperature during the measurements, the external cryostat equipment Cryosync from QuantaTech (Boynton Beach, FL, USA) was used. 

The specific surface area (SSA) was calculated by the multi-point Brunauer–Emmet–Teller (BET) method, following the BET consistency criteria of the International Organization for Standardization (ISO 9277:2010) as well as by the Quenched Solid Density Functional Theory (QSDFT) method using the Ar-carbon equilibrium transition kernel at 87.3 K for slit pores [23]. The total pore volume (TPV) and the PSD were calculated using the QSDFT method.

### 2.6. X-ray Diffraction (XRD)

XRD experiments were performed with a Bruker D8 Advance Eco (Karlsruhe, Germany) instrument using a Cu-Kα X-ray tube (λ = 0.154 nm) and an energy sensitive detector (LYNXEYE-XE). The recorded angular range (2θ) was 10–130° with a step size of 0.01° and an exposure time of 1 s step^−1^. The measurement was, as before, performed using a degassed AC—CSS sample on a zero-background sample holder (Sil’tronix Silicon Technologies, Archamps, France). To eliminate the effect of air scattering, identical conditions were used to measure the empty sample holder. The background signal was subtracted using splines. With subsequent fitting of the (10)-reflection using a Lorentzian function in combination with the Scherer Equation (1) the average crystallite size L_a_ was calculated.

(1) $$L_{a}=\;K\frac{2\pi }{\mathrm{FWHM}}$$

with the shape factor K = 1.84 [24,25,26].

The lattice parameter *a* can be calculated using following Equation (2),

(2) $$a=\frac{4\pi }{\sqrt{3\;Q_{\left(10\right)}}}\;$$

with *Q*_(10)_ being the position of the (10) in-plane reflection.

### 2.7. Raman Spectroscopy

A Horiba Jobin-Yvon LABRAM (Kyoto, Japan), equipped with a CCD detector was used to investigate the samples. The Raman spectrum was obtained with a 514.53 nm Ar laser (8 s, 7 times). 

To obtain the crystallite size L_a_ from the Raman spectra (Equation (3)), fitting of the experimental curve is carried out. In the literature, many different functions are proposed to obtain the individual contributions of the modes [26,27,28]. In this work, Lorentzian-functions for the D-, G- and 2D-band were used, which resulted in the best fit for the experimentally observed spectra [29].

(3) La=IGID·(−12.6 nm+0.033·λL)

Matthews et al. reported an empirical relationship between the crystallite size and the peak area ratio of the D-band (I_D_) and G-band (I_G_), which is valid for the used wavelength λ_L_ being between 400 nm and 700 nm [30].

### 2.8. Elemental Analysis

Carbon, hydrogen, nitrogen, sulfur, and oxygen (CHNS/O) contents were determined with a Thermo Scientific FlashSmart Elemental Analyzer. An amount of 1.5 to 3 mg of the analyte was placed in a tin or silver cup for CHNS or O measurements, respectively. For the carbonized samples (AC—CSS), 8 to 10 mg vanadium pentoxide was added as catalyst. BBOT (2,5-Bis (5-tert-butyl-benzoxazol-2-yl) thiophene)) was used as standard for all measurements. While for CHNS the samples were combusted in an oxygen atmosphere, the O determination via pyrolysis was conducted under helium flow.

### 2.9. Electrode Preparation

AC—CSS was ground by means of mortar and pestle for several minutes. The electrode slurry comprised of ca. 90 mg of AC—CSS, 5 mg of SuperP, and 5 mg of PTFE dispersed in 5 mL of isopropanol. The mixture was stirred for 3 h at 60 °C, allowing isopropanol to evaporate. Afterwards, the material was rolled out between two spacers (400 µm) by means of a stainless-steel cylinder. After drying for 24 h at 105 °C, circular electrodes with 6 mm diameter were punched. This resulted in a basis weight load of approximately 10 mg of active material per cm^2^ electrode.

### 2.10. Supercapacitor Assembly

The supercapacitors were assembled as symmetric double layer capacitors comprising electrodes with AC—CSS and YP—80F as active materials. As electrolyte, 1 M H_2_SO_4_ with various quinones (3 mM to the respective solubility limit) was used with the AC—CSS electrodes.

### 2.11. Electrochemical Characterization

Electrodes with equal weight (±0.07 mg) were paired and fixed with the conductive adhesive (EDAG PF 407C) onto the stainless-steel bolts, which were further assembled in a two-electrode configuration in a Swagelok^®^ T-cell, divided by a glass fiber separator. Prior to tightening the cell, electrolyte (200 µL) was added. The results were normalized to the respective mass of AC. Before testing, the cells were short-circuited for 15 min [31].

Long term measurements were performed on a 16 channel Arbin LBT21084 potentiostat (College Station, TX, USA). The specific current with respect to the AC in one electrode was 2 A g^−1^. The supercapacitor was charged and discharged to 1 and 0 V, respectively, for 10,000 cycles. For all other measurements a 1 channel Biologic SP-150 potentiostat (Seyssinet-Pariset, France) was used. Cyclic voltammetry (CV) was conducted with various scan rates from 2 to 100 mV s^−^^1^ between 0 V and 1 V. Galvanostatic charge and discharge (GCD) measurements were performed by applying a current of 0.1, 0.3, 1, 2, 3, 5, and 10 A g^−^^1^, charging to 1 V and discharging to 0 V. Each cycle at a specific scan rate and specific current for CV and GCD, respectively, was repeated for 20 times in subsequent order: 2, 5, 10, 20 mV s^−1^, 0.1, 0.3, 1, 2 A g^−1^, 50, 100 mV s^−1^, 3, 5, and 10 A g^−1^. All related graphs plotted and capacitances calculated derive from the last cycle of every set, unless differently stated.

The specific capacitance based on CV data was calculated according to (4)

(4) $$C_{\mathrm{CV}}=\frac{1}{2\;\Delta U\;v}\int I\;\mathrm{dU}$$

where C is the capacitance for the whole cell, ∆U is the potential range, v is the scan rate and I is the current [32,33].

The capacitance from GCD experiments (C_GCD_), was calculated according to Equation (5). As stated in Equation (6), dU/dt is given by the slope of the discharge curve until half of the maximum voltage. Since most supercapacitors in real world applications are used in a range up to half of the possible voltage (U_max_), this range (U_max_–0.5 U_max_) is also used for the calculation, with t being the time [34].

(5) $$C_{\mathrm{GCD}}=\frac{I}{\frac{\mathrm{dU}}{\mathrm{dt}}}$$

(6) $$\frac{\mathrm{dU}}{\mathrm{dt}}=\frac{U_{\max}-0.5\;U_{\max}}{\Delta t}$$

The specific capacitance for one electrode (C_sp,e_) is calculated via Equation (7).

(7) $$C_{\mathrm{sp},e}=4\;\frac{C}{m}$$

C is the respective capacitance from CV or GCD and m is the mass of active material in the whole cell. The factor of 4 compensates the mass of both electrodes and considers that the electrodes are connected in series. All capacitance-values given in this work refer to C_sp,e_. For conversion to the whole cell, a factor of 0.25 must be applied [33].

Energy density E (8) and power density P (9) were calculated according to the following Equations

(8) $$E=\frac{1}{2}\;C\;\Delta U2\;$$

(9) $$P=\frac{E}{\Delta t}$$

where Δt is the discharging time [32,33].

## 3. Results and Discussion

### 3.1. Thermal Conversion of Coffee Silver Skins 

**Figure 1:**
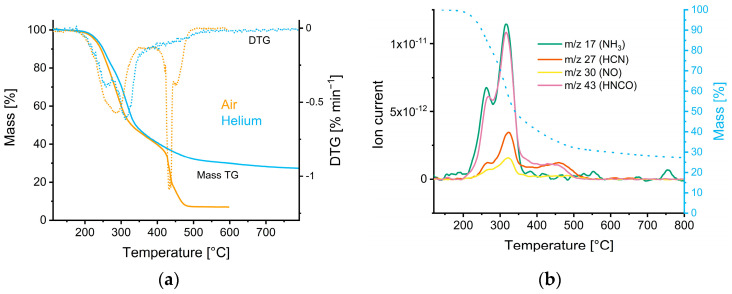
(**a**) TG (straight lines) and DTG (dotted lines) curves of a TG experiment under air as well as inert conditions in helium. (**b**) Evolution of nitrogen containing gases during the pyrolysis under helium of CSS; the dashed line indicates the mass loss of the solid material.

The conversion kinetics of biomass starting materials into carbonaceous materials are scarcely investigated. The complexity of the reactions and the availability of only selected methods to monitor the conversion limits the amount of available data in this respect. Simultaneous thermal analysis is a powerful tool to assess decomposition reactions over a wide temperature range. The thermogravimetric (TG) curve of CSS (Figure 1a) shows the mass loss in different atmospheres (blue: helium, orange: synthetic air). The 1st derivative of the TG-curves (DTG, dashed lines) indicates turning points and thus visualizes peaks of high degradation within a certain time. The thermal degradation of CSS under inert conditions (pyrolysis) takes place in two main steps, with its peak around 260 and 312 °C. Previous studies suggest that those peaks can mainly be attributed to the contribution of hemicellulose and cellulose decomposition [35,36,37], while the degradation of lignin is known to be a slow and steady process happening from 160 °C up to 900 °C [37]. A similar degradation-behavior of CSS can be observed under oxidative conditions (combustion), with an increased mass loss towards higher temperatures. The main difference between pyrolysis and oxidative combustion is the lack of char formation for the latter conditions.

As indicated by the nitrogen released compounds during pyrolysis (Figure 1b), the degradation of proteins predominantly takes place between 260 °C and 480 °C. The evolving gases are mostly related to NH_3_, HCN, NO, and HNCO as proven by mass spectrometry (m/z 17, 27, 30, and 43, respectively) [38,39,40,41]. The generation of these gaseous compounds is temperature dependent. NH_3_ and NO are nearly exclusively detected at temperatures between 260 and 350 °C, respectively, while HCN and HNCO formation was observed also at higher temperatures (up to 480 °C). The temperature-associated mass spectrometry data of CO, CO_2_, and H_2_O during pyrolysis are shown in Figure S2.

### 3.2. Characterization of CSS and AC—CSS

#### 3.2.1. Morphology and Porosity

**Figure 2:**
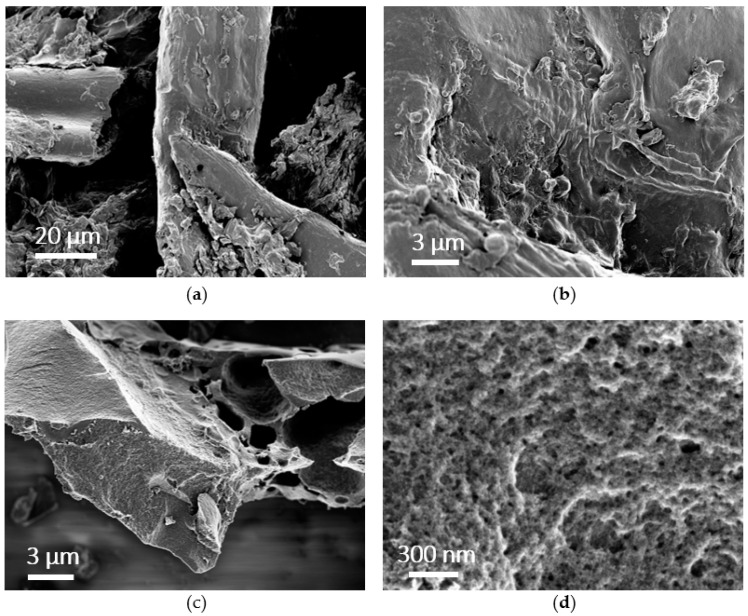
SEM images of untreated CSS at different magnifications (**a**,**b**) and AC—CSS (**c**,**d**) derived therefrom.

The morphology of CSS revealed by SEM (Figure 2a,b) appeared inhomogeneous and fibrous, while the respective AC—CSS (Figure 2c,d) showed a uniform structure. At higher magnification (Figure 2d) the presence of meso- and macropores of AC—CSS is evident. The pore size distribution (PSD), of macro-, meso-, and micropores plays a decisive role for the performance of supercapacitors. Macropores and large mesopores ensure fast charge transfer and a high transport rate to the smaller mesopores and micropores, which in turn provide high surface area for adsorption of the ions [42]. For the supercapacitor performance, it is important that the active material provides a high share of micropores approaching the radius of the solvated ion and below [43]. In addition, the presence of mesopores ensures a good route for fast electrolyte transport and thus supports higher power densities [44]. For aqueous SC systems, an approximation to the optimal mean pore size was reported to be around 0.7 nm [45]. However, it must be noted that this is strongly dependent on the ion sizes of the respective electrolyte.

**Figure 3:**
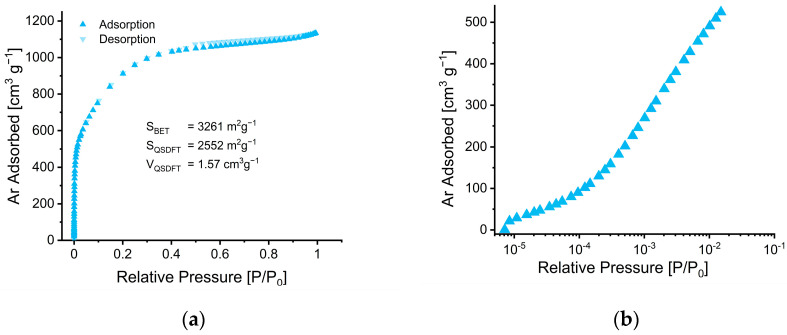

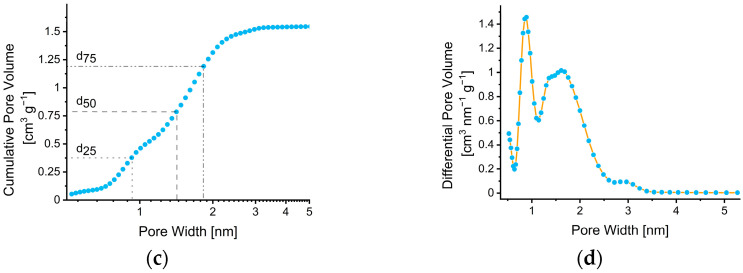
(**a**) Ar gas adsorption (triangle symbol facing up) and desorption (triangle symbol facing down) isotherms collected at 87.3 K for the degassed AC—CSS material and (**b**) the semi-logarithmic plot of the adsorption isotherm highlighting the micropore filling up to 10^−1^ P/P_0_. (**c**) Cumulative pore volume versus pore width and (**d**) differential pore volume versus pore width plots derived by the QSDFT carbon slit-pore model for Ar adsorption at 87.3 K.

The Ar adsorption/desorption isotherms at 87.3 K for the degassed carbon material are presented in Figure 3a with a linear relative pressure scale, while Figure 3b with its logarithmic *x*-axis highlights the low-pressure adsorption behavior. The AC—CSS material shows a type I isotherm, typical for microporous solids (i.e., with pore widths below 2 nm), according to the classification of the International Union of Pure and Applied Chemistry (IUPAC) [46]. The small hysteresis loop between the adsorption and desorption branches at P/P_0_ > 0.43 is associated with capillary condensation within mesopores (i.e., pore sizes of 2–50 nm). The presence of a further increase in Ar uptake at P/P_0_ > 0.94 is due to condensation in macropores (>50 nm) or adsorption onto external surfaces. The steep uptake at low relative pressures (P/P_0_ < 10^−1^) suggests micropores to be the dominating class of pores [47].

The calculated BET area, QSDFT area (more than 2500 m^2^ g^−1^) and QSDFT pore volume are shown in Figure 3a,c,d show the analysis based on the QSDFT method for the cumulative and differential pore size distributions of the materials, respectively. The volume-weighted median of the pore size (d_50_), estimated by the cumulative PSD plots (Figure 3c), is 1.42 nm, whereas the corresponding d_25_ and d_75_ values are 0.94 and 1.82 nm, respectively. The differential PSD plots in Figure 3d contain a sharp peak at 0.87 nm, followed by a broader peak located at around 1.5 nm and a small hump around 3 nm, which might be associated with the hysteresis loop.

The PSD and SSA of AC—CSS do indeed show a favorable profile for supercapacitor applications. Nevertheless, it should be noted that various parameters, such as the composition of the precursor material as well as the processing parameters have a decisive impact on the properties of the resulting AC. For this reason, a direct comparison to other biomaterial precursors is not particularly meaningful. However, to get a good overview of different starting materials and activation methods, the reader is referred to selected articles [48,49,50].

#### 3.2.2. Structural Properties

**Figure 4:**
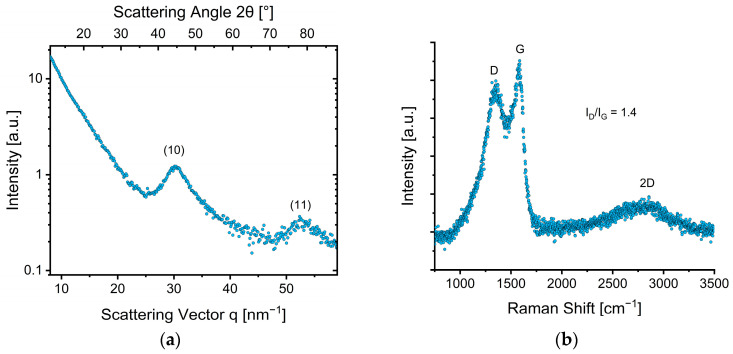
(**a**) X-ray diffractogram and (**b**) Raman spectrum of AC—CSS.

The X-ray diffractogram of the degassed powder sample (Figure 4a) shows the absence of the (002) out-of-plane stacking reflection (around q ~ 18 nm^−1^) [25] indicating the turbostratic nature of the material (high distortion of graphene sheets). The in-plane correlation length was calculated to be 2.1 nm. The (10)-in-plane-reflection around q ~ 30 nm^−1^ exhibits a broad peak, where the width correlates to the in-plane-distortions of the few stacked graphene sheets, which gives a value of *a* = 0.24 nm. 

The Raman spectra (Figure 4b) shows three characteristics of turbostratic carbon, i.e., the defect-activated D-mode (1344 cm^−1^), the G-mode (1604 cm^−1^) originating from the relative motion of the carbon atoms, and the 2D mode (2887 cm^−1^) [26,27,51].

Using the obtained peak areas from the Lorentzian fits, L_a_ was determined to be 3.1 nm. The crystallite size calculated from the Raman spectra is slightly higher than the one calculated from the XRD data, which might be attributed to the used functions, their shape, and starting conditions of the fitting procedure, as this influences the I_G_/I_D_ ratio. Therefore, we want to point out that this value must be considered with caution and should be seen more as semiquantitative measure to compare to the crystallite size derived from XRD. The 2D-band could only be fitted with one broad Lorentzian-function, which is attributed to randomly distorted stacking of the few graphene layers along the c-axis [27].

#### 3.2.3. Chemical Composition

The CHNS/O provides quantitative information on the degree of carbonization and whether nitrogen is preserved in the materials after pyrolysis. The CSS contains inorganic impurities, which is corroborated by the high ash content (7.4%). Inorganic impurities could be qualitatively confirmed in AC—CSS by SEM-EDX measurements (see Figure S1).

CSS shows a classic elementary distribution for carbohydrates (Table 1). The nitrogen-content can be attributed mainly to proteins and minor amounts of caffeine and acrylamide [6]. A factor of 6.25 applied to the nitrogen-content of CSS allows rough approximation of the protein content to 18%, which agrees with other reports [5,6].

As indicated by Figure 1b, nitrogen-containing gases are formed during pyrolysis. The decrease of the N/C ratio from CSS to AC—CSS (Table 1) suggests an over-proportional loss of nitrogen in respect to carbon.

### 3.3. Supercapacitor Performance

**Figure 5:**
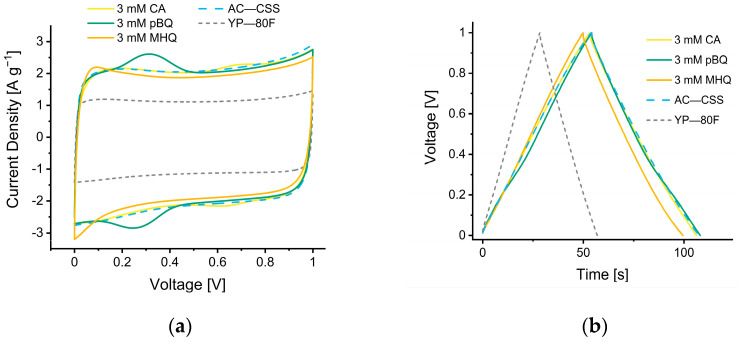

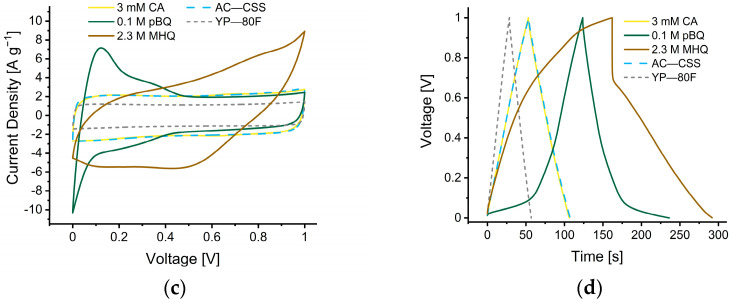
Electrochemical measurements of quinone reinforced supercapacitors compared to their reference (AC—CSS) and the electrode standard (YP—80F). (**a**) Cyclic voltammogram at 20 mV s^−^^1^, (**b**) galvanostatic charge–discharge curves at 2 A g^−1^ with the addition of 3 mM quinones, (**c**) cyclic voltammogram at 20 mV s^−^^1^, and (**d**) galvanostatic charge–discharge curves at 2 A g^−1^ with the addition of quinones at their individual solubility limit. The short dashed grey and dashed blue line indicate the standard electrode YP—80F and the quinone free reference AC—CSS, respectively. The continuous lines show the impact of quinones added to the electrolyte. Due to the fast capacitance decay of SC with 2.3 M MHQ, those curves are representative from the 6th (CV) and 12th (GCD) cycle after test start.

The AC—CSS as well as a commercial standard material (YP—80F) were assembled into symmetric supercapacitors, which have been electrochemically characterized in a two-electrode configuration. Both materials were processed in the same manner into free-standing electrodes with a mass loading of ca. 10 mg cm^−2^ using 1 M H_2_SO_4_ as electrolyte. The CVs and GCD measurements of the SCs demonstrate that the AC—CSS has a much higher capacitance than the commercial YP—80F material (75% higher at 2 A g^−1^; 188 vs. 108 F g^−1^), regardless of whether quinones were added. The shape of the CVs of the AC—CSS based SCs slightly deviates from rectangular shape, indicating a faradaic contribution [52]. In contrast, SCs from YP—80F electrodes show a typical quasi-rectangular shape, indicating primarily capacitive contributions. The addition of CA at its solubility limit in the electrolyte (3 mM) leads to small pseudocapacitive effects (Figure 5a). Other quinones investigated in this respect (para-benzoquinone, pBQ, 2-methoxyhydroquinone, MHQ) showed similar behavior as CA at the same concentration. The galvanostatic measurements showed a nearly ideal triangular shape for all the materials with only slight deviations for SCs with quinone added to the electrolyte (Figure 5b). 

By increasing the concentration to the solubility limits of pBQ and MHQ, to 0.1 and 2.3 M, respectively, different effects were observed. MHQ provides a strong faradaic contribution (Figure 5c) to the AC—CSS and furthermore exhibits strong deviation from ideal rectangular shape at a scan rate of 20 mV s^−1^. This indicates serial and parallel resistance from the electrode and electrolyte, respectively [53], and can be rationalized by a high quantity of quinones grafting onto the carbon surface that decreases the pore-accessibility [54]. This deposition may also originate from MHQ decomposition products that are redox active. This behavior is also reflected in the galvanostatic measurements that showed significant deviation from an ideal triangular shape (Figure 5d).

**Figure 6:**
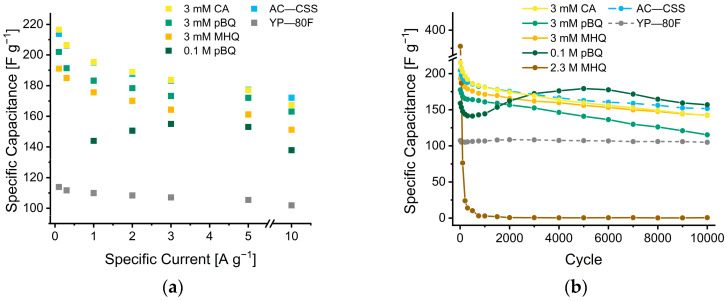
(**a**) Electrochemical characterization of AC—CSS with 1 M H_2_SO_4_ as electrolyte, with optional addition of CA, pBQ, and MHQ at various scan rates. (**b**) Long term cycling of AC—CSS with optional addition of CA, pBQ, and MHQ at 2 A g^−1^.

Further, we evaluated the influence of the charge–discharge rates on SC capacitance in a range from 0.1 to 10 A g^−1^ (Figure 6a). The depicted measurement-points are taken from the aforementioned set measurement series and thus the cycle-dependency should be taken under consideration for all values. Nevertheless, the AC—CSS clearly outperforms YP—80F over the investigated current density range (214 to 172 vs. 111 to 102 F g^−1^). The effect of quinones on the capacitance of the AC—CSS is relatively small and in most scenarios below the one of the neat AC—CSS. Only at low to moderate current densities (0.1 to 3 A g^−1^), does the addition of 3 mM CA result in a slight increase of capacitance. At higher current densities (5 and 10 A g^−1^), the presence of quinones has detrimental effects as they may slow down reaction kinetics and cause diffusive limitations. This behavior agrees with the data derived from long term measurements, where the performance of SCs equipped with quinones decreased to a higher extent after 10,000 cycles, compared to the ones of the neat AC—CSS (Figure 6b). While the capacitance of YP—80F retains 98% of its capacitive character over 10,000 cycles (Figure S3), AC—CSS electrodes just retain 75%. A contrary picture is shown by 0.1 M pBQ: first it seems to follow the trend of all AC—CSS electrodes with decreasing capacitance by approximately 10% over the first 1000 cycles. At about this value the capacitance starts to increase again; this could be caused by the formation of an electrochemically active layer at the electrode surface which might on one hand block pores. On the other hand, it could improve the electrochemical behavior of the electrode surface. After 500 cycles, AC—CSS supercapacitors with 2.3 M MHQ barely retained 5% of their initial capacitance. This can be associated with the precipitation of decomposition products at this pH value, as described elsewhere [21]. Considering a hierarchical pore size distribution, this can lead to blockages of bigger pores, thus decreasing accessibility for the electrolyte towards smaller pores, which subsequently decreases the surface area for double layer capacitance [55]. The results of all measurements are summarized in Tables S2 and S3. Furthermore, a Ragone plot (Figure S4) for measurements at 2 A g^−1^ can be found in the Supplementary Materials.

## 4. Conclusions

We demonstrated that industrial side streams from coffee production serve as a valuable source for high performance aqueous supercapacitors and quinones extracted therefrom were able to improve capacitance. Activated carbon with a specific surface area of more than 2500 m^2^ g^−1^ possess a high volumetric share of micropores smaller than the diameter of solvated SO_4_^2-^ ions to efficiently accommodate ions during charging.

Even though the solubility of caffeic acid in 1 M H_2_SO_4_ is, at 3 mM, rather low, a slight increase in capacitance (to 189 F g^−1^ at 2 A g^−1^) could be demonstrated. On the other hand, the presence of para-benzoquinone and 2-methoxyhydroquinone at various concentrations led to a decrease in capacitance. Nevertheless, the use of activated carbon from coffee silver skins as electrode material showed a 75% higher capacitance (at 2 A g^−1^) in respect to the commercial standard YP—80F. Capacitance could be improved by more than 80% by means of 2-methoxyhydroquinone, especially due to its solubility level, compared to pure activated carbon from coffee silver skin electrodes (at 2 A g^−1^), with the downside of comparably low cyclability, reducing its capacitive behavior towards zero after several hundred cycles. The addition of quinones generally caused a decrease in capacitance retention after several thousand cycles, compared to supercapacitors without quinones. The cycling stability of the quinones, as well as the coffee silver skin activated carbon, should be considered in detail in follow-up studies. Furthermore, other stable quinones should be considered and tests should be carried out with various concentrations. If those current challenges are overcome, we are optimistic that new applications for all-coffee supercapacitors will emerge.

## Figures and Tables

**Table 1:** Elemental composition (excluding ash) of CSS and AC—CSS. (n.d.: not detected).

Element	CSS [%]	AC—CSS [%]
Carbon	48.6 ± 0.3	89.2 ± 0.2
Oxygen	38.6 ± 0.3	6.7 ± 0.01
Hydrogen	6.1 ± 0.1	0.2 ± 0.01
Nitrogen	2.9 ± 0.2	0.1 ± 0.01
Sulfur	n.d.	n.d.
N/C	0.060	0.001

## Supplementary Materials

The following supporting information can be downloaded at: https://www.mdpi.com/article/10.3390/nano12152647/s1, Figure S1: EDX spectrum of AC—CSS, with an elemental map and the respective share of elements detected Figure S2: Evaluation of the most common gases during biomass pyrolysis under inert conditions, Figure S3: Capacitance retention over 10000 cycles, Figure S4: Ragone plot based on GCD-data. Due to the strong cycle dependence of 2.3 M MHQ, this meas-urement was not included, Table S1: Weight of the individual electrodes used for the respective measurements, Table S2: Capacitances at different current densities, Table S3: Capacitances at different scan rates.
