# Supplementary material for: Nanoporous Carbon Electrodes Derived from Coffee Side Streams for Supercapacitors in Aqueous Electrolytes
Source: Nanomaterials (Basel). 2022 Aug 1;12(15):2647. doi: 10.3390/nano12152647 (PMC9370518; doi:10.3390/nano12152647)
Supplement: Supplementary file 1 [file nanomaterials-12-02647-s001.zip › nanomaterials-1806464-supplementary.pdf]

nanomaterials

MDPI

Supplementary Materials

Nanoporous Carbon Electrodes Derived from Coffee Side Streams for Supercapacitors in Aqueous Electrolytes

Julian Selinger [1,2], Sebastian Stock [3], Werner Schlemmer [1], Mathias Hobisch [1], Nikolaos Kostoglou [4], Qamar Abbas [5,6], Oskar Paris [3], Christian Mitterer [4], Michael Hummel [2] and Stefan Spirk [1,*]

[1] Institute of Bioproducts and Paper Technology, Graz University of Technology, Inffeldgasse 23, 8010 Graz, Austria; julian.selinger@tugraz.at (J.S.); werner.schlemmer@ecolyte.at (W.S.); mathiashobisch@yahoo.com (M.H.)
[2] Department of Bioproducts and Biosystems, Aalto University, P.O. Box 16300, 00076 Aalto, Finland; michael.hummel@aalto.fi
[3] Institute of Physics, Montanuniversität Leoben, Franz-Josef-Straße 18, 8700 Leoben, Austria; sebastian.stock@unileoben.ac.at (S.S.); oskar.paris@unileoben.ac.at (O.P.)
[4] Department of Materials Science, Montanuniversität Leoben, Franz-Josef-Straße 18, 8700 Leoben, Austria; nikolaos.kostoglou@unileoben.ac.at (N.K.); christian.mitterer@unileoben.ac.at (C.M.)
[5] Institute for Chemistry and Technology of Materials, Graz University of Technology, Stremayrgasse 9, 8010 Graz, Austria; qamar.abbas@tugraz.at
[6] Institute of Chemistry and Technical Chemistry, Faculty of Chemical Technology, Poznan University of Technology, Berdychowo 4, 60965 Poznan, Poland
* Correspondence: stefan.spirk@tugraz.at

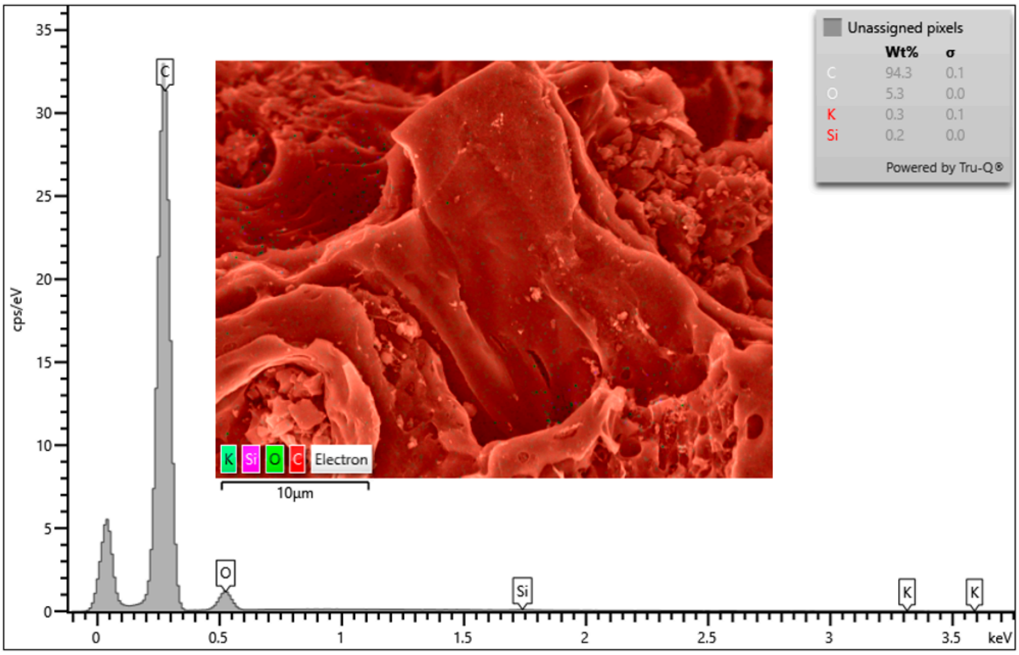

Figure S1. EDX spectrum of AC—CSS, with an elemental map and the respective share of elements detected.

Table S1. Weight of the individual electrodes used for the respective measurements.

	CV and GCD				Long Term cycling			
	Electrode weight		Respective AC		Electrode weight		Respective AC	
	Anode [mg]	Cathode [mg]	Anode [mg]	Cathode [mg]	Anode [mg]	Cathode [mg]	Anode [mg]	Cathode [mg]
YP—80F	4.18	4.27	3.76	3.84	4.26	4.26	3.83	3.83
AC—CSS	3.11	3.12	2.80	2.81	3.26	3.25	2.93	2.93
3 mM CA	3.24	3.25	2.92	2.93	3.2	3.18	2.88	2.86
3 mM pBQ	3.34	3.32	3.01	2.99	3.04	3.03	2.74	2.73
3 mM MHQ	3.40	3.35	3.06	3.02	3.07	3.06	2.76	2.75
0.1 M pBQ	2.99	2.96	2.69	2.66	2.80	2.80	2.52	2.52
2.3 M MHQ	3.03	3.1	2.73	2.79	2.82	2.73	2.54	2.46

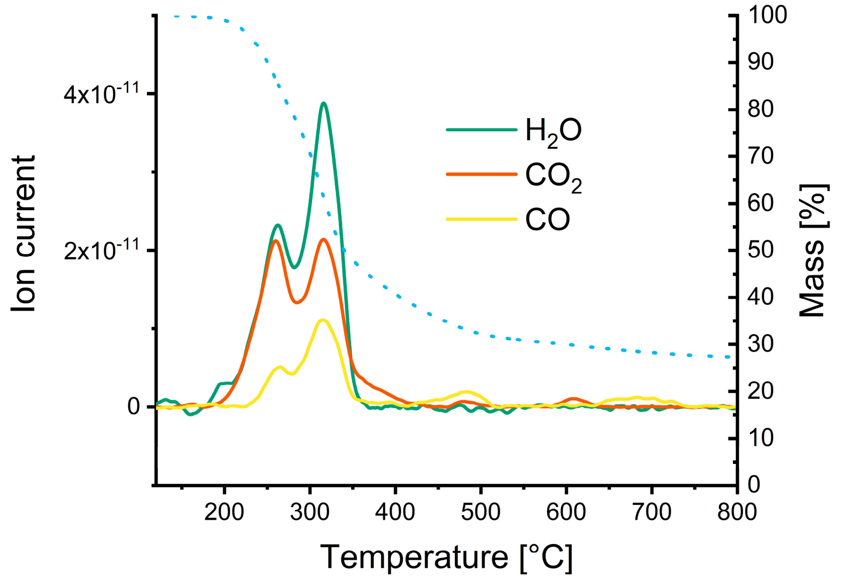

Figure S2. Evaluation of the most common gases during biomass pyrolysis under inert conditions.

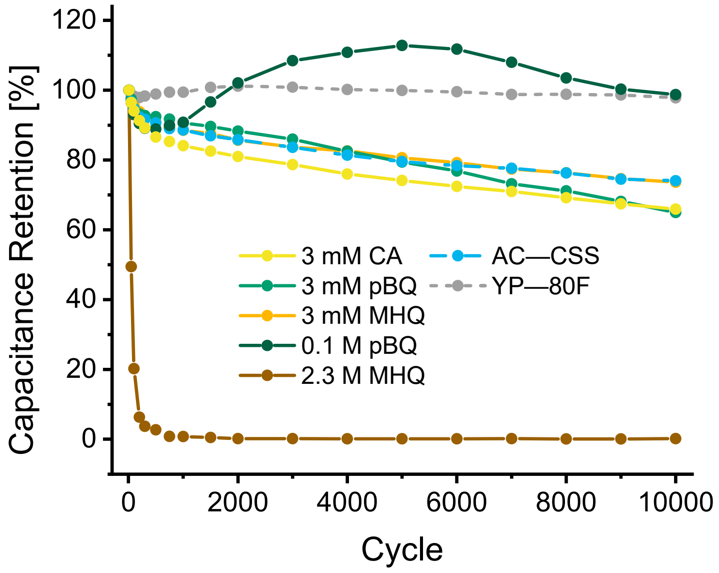

Figure S3. Capacitance retention over 10000 cycles.

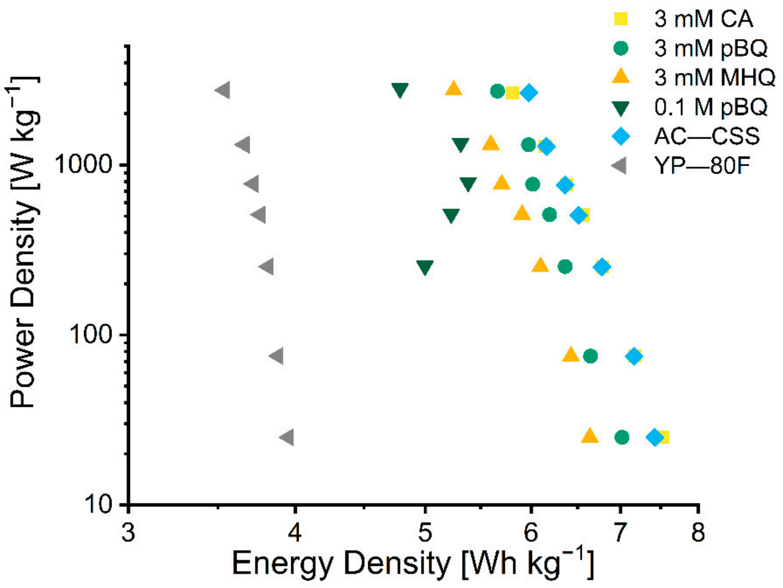

Figure S4. Ragone plot based on GCD-data. Due to the strong cycle dependence of 2.3 M MHQ, this measurement was not included.

Table S2. Capacitances at different current densities.

**Current Density**	**YP80F**	**AC—CSS**	**3mM CA**	**3mM pBQ**	**3mM MHQ**	**0.1 M pBQ**	**2.3 M MHQ ***
[A g^{-1}]	[F g^{-1}]	[F g^{-1}]	[F g^{-1}]	[F g^{-1}]	[F g^{-1}]	[F g^{-1}]	[F g^{-1}]
0.1	113.8	213.8	216.5	202.0	191.1	-- **	-- **
0.3	111.7	206.2	206.5	191.4	185.0	-- **	433.2
1.0	109.9	195.1	195.4	183.3	175.6	144.0	151.6
2.0	108.4	187.6	188.9	178.4	170.1	150.6	< 1
3.0	107.1	183.2	183.7	173.3	164.2	155.1	< 1
5.0	105.5	177.4	177.1	172.1	161.2	153.1	< 1
10.0	101.9	172.1	167.4	163.1	151.2	137.9	< 1

* 2.3M MHQ shows a strong cycle dependence thus values should be taken with caution
** charging times were exceeding one hour

Table S3. Capacitances at different scan rates.

Scan Rate	YP—80F	AC—CSS	3mM CA	3mM pBQ	3mM MHQ	0.1 M pBQ	2.3M MHQ *
[mV s^{-1}]	[F g^{-1}]	[F g^{-1}]	[F g^{-1}]	[F g^{-1}]	[F g^{-1}]	[F g^{-1}]	[F g^{-1}]
2	125.3	243.8	242.4	250.7	228.1	261.7	456.7
5	121.3	232.4	230.9	237.2	216.4	280.5	306.8
10	118.5	223.4	221.6	227.5	207.0	268.1	207.5
20	115.8	214.0	211.7	218.0	196.3	245.6	130.9
50	112.2	198.3	196.0	200.3	180.5	237.9	22.5
100	107.6	185.6	181.7	184.1	164.8	205.7	11.4

* 2.3M MHQ shows a strong cycle dependance thus values should be taken with caution
